# Yeast UBL-UBA proteins have partially redundant functions in cell cycle control

**DOI:** 10.1186/1747-1028-1-28

**Published:** 2006-12-04

**Authors:** Laura A Díaz-Martínez, Yang Kang, Kylie J Walters, Duncan J Clarke

**Affiliations:** 1 Department of Genetics, Cell Biology and Development, University of Minnesota, 6-160 Jackson Hall, 321 Church Street SE, Minneapolis, USA; 2 Department of Biochemistry, Molecular Biology and Biophysics, University of Minnesota, 6-155 Jackson Hall, 321 Church Street SE, Minneapolis, USA

## Abstract

**Background:**

Proteins containing ubiquitin-like (UBL) and ubiquitin associated (UBA) domains have been suggested to shuttle ubiquitinated substrates to the proteasome for degradation. There are three UBL-UBA containing proteins in budding yeast: Ddi1, Dsk2 and Rad23, which have been demonstrated to play regulatory roles in targeting ubiquitinated substrates to the proteasome for degradation. An involvement of these proteins in cell cycle related events has also been reported. We tested whether these three proteins act redundantly in the cell cycle.

**Results:**

Here we show that the UBL-UBA proteins are partially redundant for cell cycle related roles. *RAD23 *is redundant with *DDI1 *and *DSK2*, but *DDI1 *and *DSK2 *are not redundant with each other and the triple deletion shows a synthetic effect, suggesting the existence of at least two roles for *RAD23 *in cell cycle control. The *rad23Δddi1Δdsk2Δ *triple deletion strain delays both in G2/M-phase and in mid-anaphase at high temperatures with duplicated spindle pole bodies. Cell cycle progression in the triple deletion strain can only be partially rescued by a *rad23 *allele lacking the c-terminal UBA domain, suggesting that *RAD23 *requires its c-terminal UBA domain for full function. In addition to their ability to bind ubiquitin and the proteasome, the UBL-UBA proteins also share the ability to homodimerize. Rad23 and Dsk2 dimerization requires their UBL and/or UBA domains whereas Ddi1 dimerization does not. Here we show that Ddi1 homodimerization is necessary for its cell cycle related functions.

**Conclusion:**

The three yeast UBL-UBA proteins have partially redundant roles required for progression through mitosis.

## Background

The ubiquitin-proteasome pathway is a complex protein degradation system that is conserved from yeast to mammals and plays an important role in many processes such as cell cycle control, endocytosis and DNA repair [1-5]. In *Saccharomyces cerevisiae *Rad23, Ddi1 and Dsk2 are the three UBL-UBA proteins, which are hypothesized to shuttle ubiquitinated substrates to the proteasome for degradation [6-11] due to their ability to interact with the proteasome through their UBL domains [12-15] as well as with ubiquitin and polyubiquitinated substrates through their UBA domains [7,8,16-18]. Consistent with the shuttling hypothesis, downregulation of human *RAD23 *(hHR23) using siRNA induces accumulation of p53 which is known to be continuously degraded by the ubiquitin-proteasome pathway [19]. In addition, yeast Rad23 is sufficient for docking of ubiquitin conjugates to proteasomes isolated from a strain carrying a mutation in the ubiquitin-interacting-motif (UIM) of the proteasome subunit Rpn10 [20], which is thought to act as a receptor that binds ubiquitinated substrates. Furthermore, proteasomes isolated from a *rad23Δdsk2Δ *strain are defective in their association with endogenous ubiquitin conjugates and this defect is stronger than the one observed in *rpn10 *mutants that are unable to bind ubiquitin, suggesting that the UBL-UBA proteins are more active than Rpn10 in delivery of ubiquitinated substrates to the proteasome [20].

Interestingly, none of the UBL-UBA proteins are essential for viability. Single deletion of *RAD23 *or *DSK2 *induces partial stabilization of a model degradation-substrate whereas the *rad23Δdsk2Δ *double deletion completely stabilizes the substrate [7] and accumulates endogenous polyubiquitinated proteins at high temperatures [21], suggesting that Rad23 and Dsk2 function redundantly in ubiquitin-mediated protein degradation. In addition to these genetic interactions, UBL-UBA proteins have been shown to physically interact, forming both homodimers and heterodimers [7,22]. Dsk2 and Rad23 homodimerization occurs through their UBL and/or UBA domains [17,23,24], whereas Ddi1 homodimerization requires neither of these domains [17]. Although, in vivo, heterodimerization of UBL-UBA proteins is likely to also occur via bridging molecules [22], direct interactions between the UBL and UBA domains promoting heterodimer formation have been demonstrated [22,23]. Rad23 homo and heterodimerization of UBL-UBA family members has been suggested to play a role in regulating their interactions with other components of the ubiquitin-proteasome pathway and to lead to the formation of multimeric complexes with polyubiquitin chains [23,25]. Such interactions could increase the targeting efficiency of ubiquitinated substrates for degradation.

One of the cellular processes that relies on ubiquitin-proteasome dependent proteolysis is cell cycle progression. Cell division is a complex process; it requires that a series of steps are fulfilled in a specific and unidirectional order. When cell cycle regulation fails genetic instability and aneuploidy often arise, which are hallmarks of and might initiate cancers [26,27]. A long list of cell cycle regulators are known to be degraded by the ubiquitin-proteasome pathway [2]. Several pieces of evidence suggest a role for UBL-UBA containing proteins in cell cycle control. In budding yeast, overexpression of Dsk2 is toxic, inducing accumulation of ubiquitinated substrates [12] and arresting the cells in mitosis with abnormal nuclear position and short bipolar spindles [28]. Ddi1 is involved in the degradation of an SCF component, the F-box protein Ufo1, involved in the G1/S transition [29], as well as one of its targets the Ho endonuclease [30]. Overexpression of *DDI1 *or *RAD23 *suppresses the temperature sensitive phenotype of a *PDS1 *mutant allele (*pds1–128*) [31]. Combined deletion of the *RAD23 *and *DDI1 *C-terminal UBAs, but not the single deletions, results in premature loss of cohesion and spindle elongation in the presence of hydroxyurea (HU), which is known to activate the S-phase checkpoint [31]. In addition, the *dsk2Δrad23Δ *double deletion, but not the single deletions, is reported to be defective in Spindle Pole Body (SPB) duplication at high temperature, inducing the formation of monopolar spindles and subsequently arresting the cells in mitosis [28]. These results suggest redundant roles for the UBL-UBA proteins in cell cycle events.

Here we demonstrate that deleting *RAD23 *in combination with either *DDI1 *or *DSK2 *induces cell cycle delays in the G2/M-phase and anaphase at high temperatures, indicating that *DDI1 *and *DSK2 *are redundant with *RAD23*. In addition, we provide evidence that *DDI1 *and *DSK2 *are not functionally redundant and therefore that *RAD23 *has at least two cell cycle-related functions. This hypothesis is supported by the synthetic effect observed in the triple deletion, confirming that *RAD23-DDI1 *redundancy is different from that of *RAD23-DSK2 *(i.e. the triple deletion shows an additive phenotype). Surprisingly however, the arrest is not due to failure in SPB duplication.

## Results

### *RAD23*, *DDI1 *and *DSK2 *have partially redundant roles in cell cycle progression

Possible roles for Rad23 and Ddi1 in the cell cycle have been suggested by their ability to rescue the temperature sensitivity of *pds1–128 *[31], while Rad23 and Dsk2 have been reported to have roles in SPB duplication and Dsk2 overexpression leads to mitotic arrest [28]. To test whether the three yeast UBL-UBA proteins have a redundant role in cell cycle progression we took a genetic approach, obtaining strains deleted for each one of the UBL-UBA genes (Table 1) as well as all the double deletion combinations and the triple deletion, and asked whether any redundancy in terms of functions in cell cycle progression could be observed.

**Table 1:** Yeast strains used in this study

1167	*MATalpha pds1–128*
1494	*pds1–128 GAL-ddi1ΔUBA [HIS]*
1493	*pds1–128 GAL-DDI1 [HIS]*
1671	*MATa bar1Δ GFP:TUB1::uRA3 ARG4*
2073	*MATalpha pds1–128 GAL-ddi1-A407L*
2074	*MATalpha pds1–128 GAL-ddi1-L426A*
L153	*MATalpha pds1–128 arg 4 GAL:ddi1Δ184–285*
L156	*MATalpha pds1–128 arg 4 GAL:ddi1Δ184–285*
L150	*MATalpha pds1–128 arg 4 GAL:DDI1 [LEU]*
L169	*MATa bar1Δ spc42:GFP [TRP]*
L182	*MATa bar1Δ dsk2::KAN spc42:GFP [TRP]*
L185	*MATa bar1Δ rad23::KAN dsk2::KAN SPC42:GFP [TRP]*
L187	*MATa bar1Δ ddi1::KAN dsk2::KAN SPC42:GFP [TRP]*
L194	*MATa bar1Δ rad23::KAN SPC42:GFP [TRP]*
L196	*MATa bar1Δ ddi1::KAN rad23::KAN SPC42:GFP [TRP]*
L201	*MATa bar1Δ ddi1::KAN rad23::KAN dsk2::KAN TUB1:GFP [URA]*
L218	*MATa bar1Δ ddi1::KAN dsk2::KAN rad23::KAN SPC42:GFP [TRP]*
L221	*MATa bar1Δ ddi1::KAN SPC42:GFP [TRP]*
L263	*MATa bar1Δ ddi1::KAN dsk2::KAN TUB1:GFP::URA3 RAD23ΔUBA2-MYC [TRP] SPC42:GFP [TRP]*

Although none of the single deletions have temperature sensitive phenotypes, the *rad23Δdsk2Δ *double deletion has been reported to accumulate ubiquitinated substrates at 37°C [21], as well as have a temperature sensitivity phenotype at 35°C [28]. Partially consistent with these results, we observed that *rad23Δ*, *ddi1Δ *or *dsk2Δ *singly deleted strains as well as *rad23Δddi1Δ *and *ddi1Δdsk2Δ *doubles were not temperature sensitive. Surprisingly, the *rad23Δdsk2Δ *double deletion was also alive at 37°C. In contrast, the *rad23Δdsk2Δddi1Δ *triple deletion was temperature sensitive at 37°C, suggesting the existence of redundant roles for Ddi1, Dsk2 and Rad23 at high temperatures.

**Figure 1:**
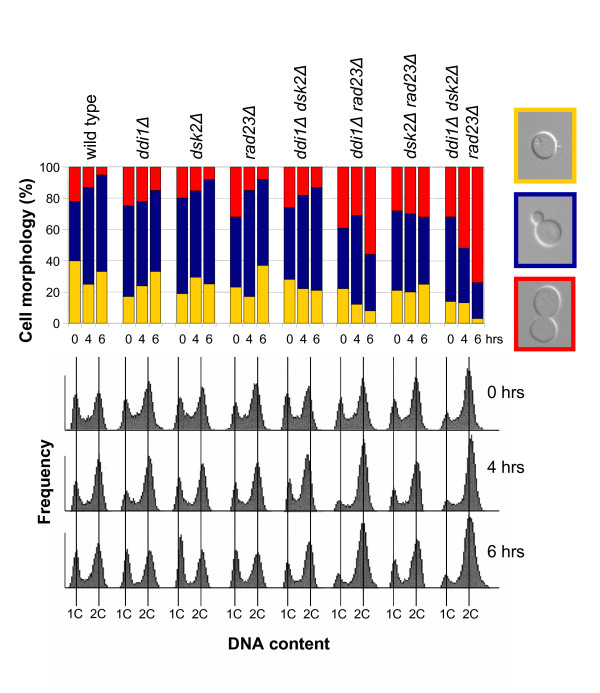
**Deletion of *DDI1*, *DSK2 *and *RAD23 *has a synthetic effect on cell cycle progression at high temperatures**. (A) Cells were grown to mid-log phase in liquid YEPD at 30°C then shifted to 37°C for 0, 4 and 6 hrs. Cell cycle distribution was determined by bud morphology: cells in G1 are unbudded (yellow), S-phase cells with small buds (blue) and G2/M cells with large buds (red). Large buds (dumbbells) are defined as cells where the bud is as big as the mother cell. In parallel, samples were taken for FACScan analysis of DNA content (lower panel).

Next we asked whether these proteins were involved in cell cycle progression. Briefly, all the combinations of single, double and the triple deletions were incubated in YEPD overnight at 30°C, diluted the next morning and incubated at 37°C. Cell cycle progression was evaluated by scoring the proportions of the different cell morphologies: unbudded cells (G1, yellow), cells with small to medium buds (S phase, blue) and dumbbells (G2/M, red) (Figure 1, upper panel). Samples were also taken for FACScan analysis of DNA content (Figure 1, lower panel). Interestingly both *rad23Δdsk2Δ *and *rad23Δddi1Δ *showed a slight accumulation of cells in G2/M, but the accumulation observed in the *rad23Δddi1Δdsk2Δ *triple deletion reached ~70% after 6 hours at the non-permissive temperature (Figure 1). On the other hand, cell cycle distribution in the *ddi1Δdsk2Δ *double deletion was not different from those of the single deletions. These results suggest that *RAD23 *is redundant with *DDI1 *and *DSK2 *but that *DDI1 *and *DSK2 *do not share a common functional role needed for timely cell cycle progression. Furthermore, these data suggest a synthetic effect, that is, *RAD23 *redundancy with *DDI1 *is different than that of *RAD23 *with *DSK2 *and the effect seen in the triple deletion is the result of both defects combined (Figure 1).

### The c-terminal UBA domain of Rad23 is necessary for its cell cycle functions

**Figure 2:**
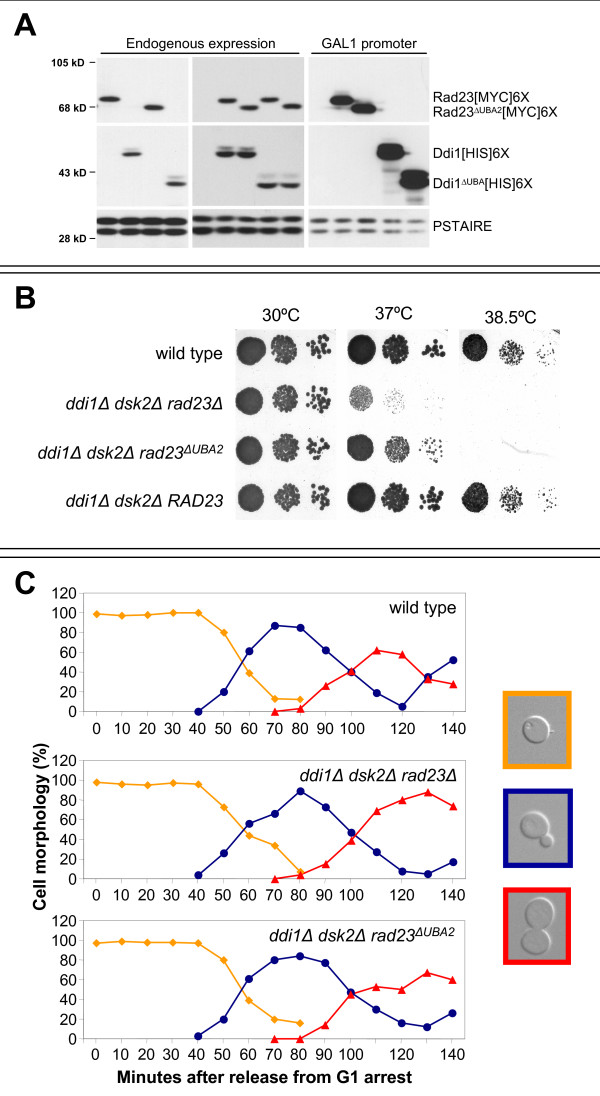
**Cell cycle arrest in cells lacking *DDI1*, *DSK2 *and the c-terminal UBA (UBA2) domain of Rad23**. (A) Western blots showing relative protein levels of Rad23, Ddi1 and mutant forms in yeast extracts. Left two panels show endogenous levels of these proteins and the right panel shows levels produced after expression of constructs from the *GAL1 *promoter. In each case, the wild type and mutant version were tagged identically at their C-termini – [MYC]6x for Rad23 and [HIS]6x for Ddi1. PSTAIRE is a loading control. Strains expressed (from left to right) – Left panel: *RAD23 [MYC]6x*, *DDI1 [HIS]6x*, *RAD23*^*ΔUBA2*^*[MYC]6x*, *DDI1*^*ΔUBA*^*[HIS]6*x; Middle Panel: no tag control, *RAD23[MYC]6x *and *DDI1[HIS]6x*, *RAD23*^*ΔUBA2*^*[MYC]6x *and *DDI1[HIS]6x*, *RAD23[MYC]6x *and *DDI1*^*ΔUBA*^*[HIS]6*x, *RAD23*^*ΔUBA2*^*[MYC]6x *and *DDI1*^*ΔUBA*^*[HIS]6*x; Right Panel: no tag control, *GAL1-RAD23[MYC]6x*, *GAL1-RAD23*^*ΔUBA2*^*[MYC]6*x, *GAL1-DDI1[HIS]6x*, *GAL1-DDI1*^*ΔUBA*^*[HIS]6*x. Upper band of Ddi1 is a phosphorylated species (data not shown). (B) Synthetic effect of UBL-UBA mutants on temperature sensitivity. The indicated strains were grown to mid-log phase and serial dilutions were spotted onto YEPD plates and incubated at the indicated temperatures for 48 hrs. Levels of Rad23 and Rad23^ΔUBA2 ^protein expression were determined as shown in A. (C) Kinetics of cell cycle progression in wild type , *ddi1Δ dsk2Δ rad23Δ *and *ddi1Δ dsk2Δ rad23ΔUBA2 *cells. (*ddi1Δdsk2Δ *strains behaved identically to the wild type control; Figure 1 and data not shown.) Cells were arrested in G1 at 30°C with alpha-factor and released in rich medium at 37°C. Cell cycle progression was monitored by bud morphology: unbudded cells (G1, yellow line), cells with small buds (S-phase, blue line) and cells with large buds (G2/M, red line).

Although both UBA domains of Rad23 have been shown to bind ubiquitin both in yeast [17] and in humans (hHR23A) [32], there is a specific requirement for its c-terminal UBA domain (UBA2) in mediating cell cycle arrest after binding to the HIV-1 Vpr protein [33]. Therefore we tested whether UBA2 is required for the cell cycle function of Rad23 by examining cell cycle progression in a strain in which the sole UBL-UBA protein is *rad23ΔUBA2 *(Figure 2). A recent study suggested that the C-terminal UBA domain of Rad23 acts as a stabilization domain [34], preventing proteasome-dependent degradation of RAd23. It was therefore important to determine if *rad23ΔUBA2 *was stable in our strains. As shown in Figure 2A, the protein levels of *rad23ΔUBA2 *and Rad23 were identical, whether these genes were expressed from their endogenous promoters or exogenously from the *GAL1 *promoter. This was also the case when we compared the protein levels of Ddi1 and *ddi1ΔUBA *(Figure 2A). After confirming that the wild type and mutant proteins were present in identical amounts in our strains, we spotted serial dilutions of the corresponding strains onto rich medium and grew at a range of temperatures for several days (Figure 2B). A partial recovery of the temperature sensitivity of the *rad23Δddi1Δdsk2Δ *triple deletion strain was observed after introducing *rad23ΔUBA2*, but full recovery was observed only when the full-length *RAD23 *gene was introduced (Figure 2B). These data suggest that, although both UBA-motifs can interact with ubiquitin [17], full Rad23 activity requires both UBA domains.

To explore this result in more detail, we examined the kinetics of cell cycle progression in strains that contained either no UBL-UBA proteins (*rad23Δddi1Δdsk2Δ*) or *rad23ΔUBA2 *(*ddi1Δdsk2Δrad23ΔUBA2*) as the sole UBL-UBA protein (Figure 2C). After release from a G1 arrest induced by alpha-factor treatment at the non-permissive temperature of 37°C, re-entry into the cell cycle and budding occurred with the same kinetics in these strains and in a wild type control. G2/M accumulation (red line) in the wild type strain peaked at 120 minutes and decreased to 28% 140 minutes after release as the cells entered the next cell cycle. Initiation of a second cell cycle was also indicated by the presence of a second wave of small-budded cells (blue line) appearing at 130 minutes. In contrast, the triple deletion strain entered G2/M with the same kinetics as wild type, but remained there even after 140 minutes (75% in G2/M). Meanwhile, *ddi1Δ dsk2Δ rad23ΔUBA2 *cells entered G2/M with the same kinetics as both the wild type and the triple deletion strains, but accumulated in G2/M, albeit to a lesser extent than the triple deletion (60% at 140 minutes). This finding suggests that *rad23ΔUBA2 *partially alleviates the delay and confirms that complete function of Rad23 requires the full-length protein and that the internal UBA domain (UBA1) can only partially alleviate the G2/M delay.

### G2/M arrest is not due to a failure in SPB duplication

**Figure 3:**
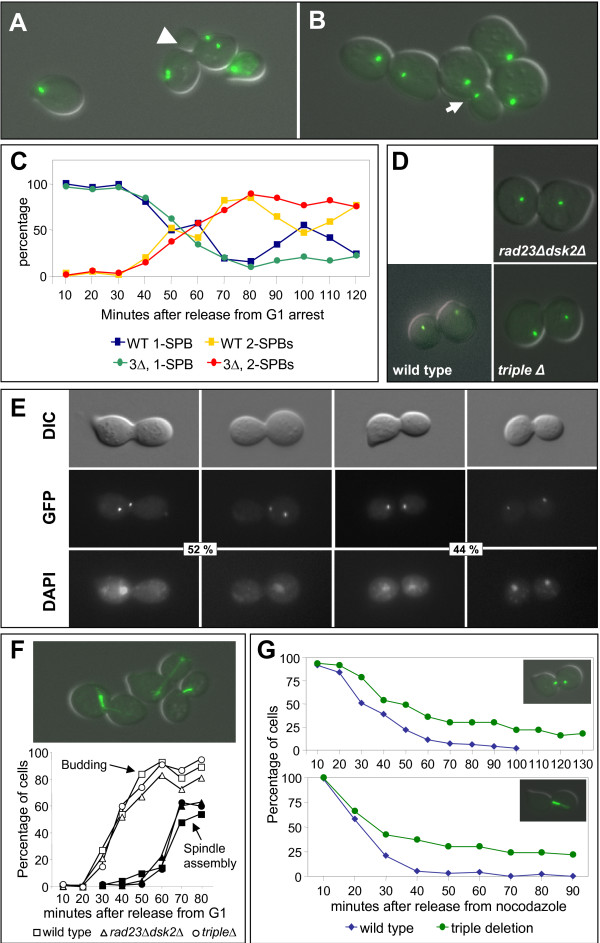
**SPB duplication and spindle function**. (A) Photomicrograph of wild type SPC42:GFP cells from time course in C, 110 minutes after alpha-factor release. Arrowhead shows rebudded cell that has already undergone a second SPB duplication. (B) Photomicrograph of *rad23Δdsk2Δddi1ΔSPC42:GFP *cells from time course in C, 110 minutes after alpha factor release. Arrow shows cell with G2-like short inter-SPB distance. (C) SPB duplication kinetics. Wild type (WT) and *rad23Δdsk2Δddi1Δ *(3Δ) cells were arrested with alpha factor and released into rich medium at 37°C. Samples were taken every 10 minutes and SPBs in each cell were counted. (D) Photomicrographs showing SPBs (*SPC42:GFP*) in wild type, *rad23Δdsk2Δ *and *rad23Δdsk2Δddi1Δ *cells at 90 minutes after release from G1 arrest at the restrictive temperature. (E) Photomicrographs showing DIC, *SPC42:GFP *signals, and DAPI staining of DNA in *rad23Δdsk2Δddi1Δ *cells at 180 minutes after release from G1 arrest at the restrictive temperature of 37°C. Left two panels show G2-like cells (52% of the large budded cells) with a single nucleus and SPBs typically separated by less than 4 μm. Right two panels show anaphase cells with divided nuclei (44% of the large budded cells) and SPBs typically separated by > 4 μm. The remaining large budded cells had stretched single nuclei. (F) Timing of bud emergence and spindle assembly after release from alpha-factor induced G1 arrest in wild type, *rad23Δdsk2Δ*, and *rad23Δdsk2Δddi1Δ *cells. Photomicrograph shows *rad23Δdsk2Δddi1ΔTUB1:GFP *cells at 37°C with both G2 and anaphase spindles. (G) Nocodazole release experiment in *rad23Δdsk2Δddi1ΔSPC42:GFP *cells. Cells were arrested with alpha factor, released into medium containing nocodazole and grown at 30°C for 2 hours, then washed and released into medium containing alpha factor to prevent re-budding upon progression to G1. Samples were taken every 10 minutes and SPBs with short G2-like inter-SPB distance (1–4 μm, inset micrograph) were counted (upper panel). This protocol was repeated in *rad23Δdsk2Δddi1Δ TUB1:GFP *cells (lower panel). Samples were taken every 10 minutes and short G2 spindles (inset micrograph) were counted.

Since defects in SPB duplication leading to G2/M arrest have been reported for strains lacking *RAD23 *and *DSK2 *[28], we asked if there is a SPB duplication defect in the triple deletion strain. To visualize SPBs in live cells we used strains expressing *SPC42:GFP *(Table 1). Wild type or *rad23Δddi1Δdsk2Δ *triple deletion strains expressing *SPC42:GFP *were arrested in G1 using alpha-factor and released into fresh medium at the restrictive temperature. Samples were taken every 10 minutes, visualized (Figure 3A–B) and scored according to SPB number (Figure 3C). Surprisingly, the triple deletion strain separated SPBs normally (Figure 3A–C). The kinetics of SPB separation in the triple deletion were indistinguishable from that of the wild type strain (Figure 3D), except that the triple deleted cells arrested as large budded cells with two SPBs (Figure 3E,D). In the wild type strain, SPB separation initiated at ~40 minutes after release from G1 at the restrictive temperature (Figure 3C, yellow line) and peaked at ~80 minutes. In accordance, the population of cells with a single SPB signal (Figure 3C, blue line) was depleted within the same timeframe. After 100 minutes a second wave of cells with a single SPB signal peaked, followed by a second wave of SPB separation as cells enter another cell cycle. A mixed population of unbudded cells with single SPB signals and re-budded cells with newly separated SPBs could be observed 110 minutes after the alpha-factor release (Figure 3A, arrow head), consistent with cells entering the next cell cycle. *ddi1Δdsk2Δrad23Δ *cells separated their SPBs with the same kinetics as wild type cells, but then delayed or arrested with large buds and two SPBs (Figure 3C, red line). A very similar phenotype was seen in *dsk2Δrad23Δ *mutants (data not shown). Interestingly, the arrest observed in the *dsk2Δrad23Δ *and the triple deleted cells was not homogeneous, since some of the cells arrested as dumbbells with well separated SPBs, resembling anaphase cells (Figure 3B, right cells, and Figure 3D), while the rest of the arrested cells possessed SPBs that were separated by a distance typical of G2 cells (Figure 3B, arrow).

DAPI staining of DNA in these arrested cells confirmed that some cells were arrested in a G2-like stage with a single nucleus (Figure 3E, left two panels), while other cells were arrested in early (3^rd ^panel) or late anaphase (rightmost panel). Arrest in G2 or anaphase could have been due to defects in spindle assembly. Therefore, to further explore these results and examine in more detail the dynamics of spindle assembly in the triple deletion, we constructed strains harboring a *TUB1:GFP *gene (Table 1). In agreement with the results observed in the *SPC42:GFP *strains, wild type cells and *ddi1Δdsk2Δrad23Δ *cells assembled their spindles with the same timing, relative to bud emergence (Figure 3F).

In a final series of experiments, we performed time courses in which cells were released from a nocodazole arrest at the restrictive temperature of 37°C (Figure 3G). The percentage of wild type cells with G2-SPBs (Figure 3G, top panel, blue line) began to decrease by 20 minutes after release from nocodazole and cells with G2-SPBs were completely depleted (i.e. the cells had undergone anaphase) 90 minutes after the release. In contrast, the triple deleted cells showed a delay in anaphase onset, as judged by the distance between the SPBs, and ~30% remained in G2 (Figure 3G, top panel, green line) at the time wild type G2-cells had been completely depleted. Similar data were obtained in strains expressing *TUB1-GFP*. Within about 10 minutes after release of the nocodazole block, almost all large budded cells had assembled G2 spindles (Figure 3G, lower panel). Wild type cells initiated spindle elongation ~20 minutes after release from nocodazole and by 40 minutes most of the cells had completed anaphase (Figure 3G, lower panel, blue line). In contrast, some *ddi1Δdsk2Δrad23Δ *cells remained in G2, as judged by their short (1–4 μM) spindles, even 90 minutes after the release (Figure 3G, lower panel, green line). Together, these results suggest *ddi1Δdsk2Δrad23Δ *cells delay cell cycle progression at two distinct points when grown at high temperatures: a fraction of the triple deleted cells delay before anaphase with duplicated SPBs but short spindles, whereas other cells delay in a mid-anaphase or late-anaphase state with partly-elongated or fully-elongated spindles. This suggests that combined deletion of *RAD23*, *DDI1 *and *DSK2 *affects spindle dynamics and cell cycle progression, but not SPB duplication.

### Ddi1 homodimerization is necessary for *pds1–128 *rescue

**Figure 4:**
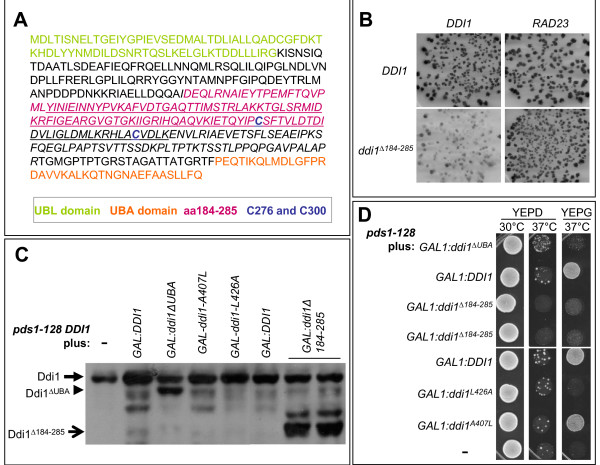
**Ddi1 homodimerization is required for rescue of *pds1–128 *temperature sensitivitity**. (A) Ddi1 sequence with the UBL and UBA domains in green and orange, respectively. Residues 184–285 are displayed in plum and C276 and C300 in blue. (B) Yeast two hybrid assay reveals that residues 184–285 are important for homodimerization of Ddi1 but not for its interaction with Rad23. Production of blue color in x-gal containing medium signals for a positive interaction between two proteins [17]. *ddi1Δ184–285 *interaction with *DDI1 *is highly reduced but *ddi1Δ184–285 *interaction with *RAD23 *is similar to that of wild type *DDI1*. (C) Western blots showing levels of different Ddi1 variants. Cells were grown on YEPR overnight, diluted 1:10 and expression of *DDI1 *constructs was induced from the *GAL1 *promoter by Galactose addition. These strains corresponded to the *pds1–128 DDI1 *strains assayed in D and expressed the indicated constructs exogenously from *GAL1 *in addition to expressing endogenous *DDI1 *(which is approximately 47 kD) from its native locus. The leftmost lane contains a vector control, not expressing *DDI1 *exogenously. The Western blot was probed with poly-clonal anti-sera against Ddi1 that recognized all of the mutant forms tested. Where wild type *DDI1 *is expressed from the *GAL1 *promoter, the upper band contains endogenous and exogenous Ddi1. (D) Rescue of *pds1–128 *temperature sensitivity. Serial dilutions of the indicated strains (corresponding to those tested in D) were spotted onto YEPD or YEPG plates and incubated for 24 hr at the indicated temperatures.

In addition to their abilities to bind ubiquitin and the proteasome, Dsk2, Ddi1 and Rad23 share the ability to form homodimers [22,24]. The importance of dimerization for the function of these proteins is not known however. Dsk2 and Rad23 homodimerization involves their UBL and/or UBA domains [17,23,24], which makes it difficult to obtain mutants that disrupt homodimerization but remain capable of binding to ubiquitin and the proteasome. Ddi1, on the other hand does not require either the UBA or the UBL domains for homodimerization [22]. Therefore we tested whether homodimerization is important for the role of Ddi1 in one specific cell cycle related event; stabilization of Pds1–128. Firstly we more precisely defined the domain important for Ddi1 homodimerization to a region encompassing residues 184 to 285 (Figure 4A, plum). By deleting this region a new *DDI1 *allele, *ddi1Δ184–285*, was obtained that was no longer able to dimerize with wild type Ddi1 but was still able to bind both Rad23 (Figure 4B) and ubiquitin (data not shown).

To ask whether Ddi1 dimerization was important for the role of Ddi1 in stabilizing Pds1–128, we overexpressed exogenous *ddi1Δ184–285 *in a *pds1–128 DDI1 *strain (Figure 4C) and asked whether this mutant could rescue the temperature sensitivity of this strain (Figure 4D). When overexpressed, neither *ddi1Δ184–285*, nor *ddi1 *alleles that disrupt UBA-ubiquitin interaction (*ddi1ΔUBA *or *ddi1-L426A *[17]) rescued the temperature sensitivity of the *pds1–128 *strain but overexpression of wild type *DDI1 *or *ddi1-A407L *(a *ddi1*-UBA mutant that still interacts with ubiquitin) did rescue the temperature sensitivity of *pds1–128*. The protein levels of wild type *Ddi1 *and the mutant forms after expression from the *GAL1 *promoter were all similar (Figure 4C). This was also the case when tagged versions of *DDI1 *and mutants were expressed from the *GAL1 *promoter (Figure 2A). Therefore, these results suggest that both homodimerization and ubiquitin interaction are required for rescue of *pds1–128 *by overproduced Ddi1.

## Discussion

The UBL-UBA proteins Ddi1, Dsk2 and Rad23 are conserved from yeast to humans but are not essential for viability in yeast, suggesting that they might perform overlapping functions. We investigated this possibility by removing all three UBL-UBA proteins to find that they are essential for viability and cell cycle progression at high temperatures. Interestingly, these analyses did not provide evidence for a functional overlap between Ddi1 and Dsk2, but rather indicate that Rad23 has redundant functions with each of Ddi1 and Dsk2. A strain deleted for all three of these genes exhibited a synthetic effect, suggesting that *RAD23 *has at least two independent roles that are required for viability at high temperatures; one of them shared with *DDI1 *and the other one with *DSK2*. Interestingly, these redundancies do not seem to be a product of evolutionary divergence of duplicated genes, since neither *RAD23, DSK2 *or *DDI1 *are elements in the duplication blocks that have been described to have arisen after the yeast genome duplication event. Furthermore, they lie next to elements from different duplication blocks: block 28 for *RAD23*, block 46 for *DSK2 *and block 13 for *DDI1 *[35], which further suggests that they are functionally related, but not evolutionarily related.

Since these proteins have been shown to be involved in cell cycle related phenomena [28,31], we examined cell cycle progression at the restrictive temperature and found that *ddi1Δdsk2Δrad23Δ *cells arrest or delay in G2/M and anaphase. Surprisingly, the arrest observed in the triple deletion was not due to a failure in SPB duplication. We were also not able to observe SPB duplication defects in the *rad23Δdsk2Δ *double deletion strains at 37°C (data not shown), as had been previously reported [28]. We currently do not know the reason for this contradictory result, but it could be due to differences in the genetic background between the BF264-*15Daub *(this study) and S288c strains [28].

Interestingly, when we examined the kinetics of spindle elongation (Figure 3) we found that the arrest observed in the triple deletion strain was not homogeneous. There were two distinct populations: cells that arrested with G2-like spindles and had a single nucleus, and cells that arrested with partly or fully elongated spindles and divided nuclei. One possibility is that the *ddi1Δdsk2Δrad23Δ *cells progressively accumulate ubiquitinated substrates that somehow interfere with degradation of other cell cycle regulatory proteins. The existence of two arrest points might therefore be a reflection of two cell cycle stages at which proteasome function is required: some of the mutant cells may have accumulated enough substrates to enforce arrest at the earlier time point whereas other cells might be able to reach the latter cell cycle stage. However, the specific reasons why subpopulations of the triple deleted cells arrest at the earlier or later time point remain to be determined.

The ability of UBL-UBA proteins to both hetero and homodimerize, as well as to bind to polyubiquitin in tandem [23] suggests the possibility that UBL-UBA dimerization regulates binding of these proteins to ubiquitinated substrates, possibly by bringing more than one UBL-UBA protein in close proximity to the ubiquitin chain [23,36]. Ddi1 is the only UBL-UBA protein in which homodimerization does not involve the UBL or UBA domains, allowing the study of the role of dimerization without affecting its interaction with ubiquitin or the proteasome. We have shown that disruption of Ddi1 homodimerization affects Ddi1's ability to rescue the *pds1–128 *temperature sensitivity, suggesting that Ddi1 homodimerization is necessary for its role in Pds1–128 stabilization. Ddi1 dimerization does not involve the cystein residues at positions 276 and 300 (Figure 4A, blue) since a C276S C300S double mutant is still able to interact with wild type Ddi1 (data not shown). Interestingly, it has been suggested that Pds1–128 stabilization by Ddi1 might be due to de-ubiquitination of Pds1–128 through the aspartyl-protease domain of Ddi1 (underlined in Figure 4A)[37]. Our results suggest that this could be possible. First, the *ddi1Δ184–285 *allele lacks half of the aspartyl-protease domain. Second, the aspartyl protease domain requires dimerization to become active, and the *ddi1Δ184–285 *allele does not dimerize, suggesting that the predicted Ddi1 aspartyl-protease activity is not functional in *ddi1Δ184–285 *and could hypothetically be involved in *pds1–128 *rescue.

## Conclusion

Redundancy of function among the budding yeast UBL-UBA proteins can be predicted based on their common abilities to bind ubiquitin and the proteasome. We have shown that the yeast UBL-UBA proteins have partially redundant functions, Rad23 being redundant with both Ddi1 and Dsk2. Surprisingly Ddi1 and Dsk2 do not share redundant functions but a synthetic phenotype is observed in the triple deletion strain in which cells become delayed in both G2 and anaphase.

## Materials and methods

### Strains and Plasmids

Strains are derivatives of BF264-*15Daub *[38], unless otherwise noted. Standard genetic procedures were used [39]. Spindles were visualized by expressing a *GFP:TUB1 *construct [40,41] integrated at the *URA3 *locus. SPBs were visualized by expressing a *GFP-SPC42 *construct [28]. Cultures were grown on YEPD at 30°C unless otherwise stated. Micrographs were acquired with a Zeiss Axioplan2 Microscope, using an alpha-Plan-FLUAR 100X/1,45 oil objective, an Axiocam HRm camera and Axiovision software.

The *pB42AD-ddi1Δ184–285 *vector was obtained by ClaI digestion of the *pB42AD-DDI1 *two-hybrid vector [17,31] followed by self-ligation. *pYIPG2-DDI1 *and *pYIG2-ddi1Δ184–285 *were obtained by PCR from the respective pB42AD-vectors. The PCR fragments were cloned into a pCR2.1-TOPO vector (Invitrogen), cut out with BamHI and cloned into the pYIPG2 vector. These constructs were subsequently integrated at the *LEU2 *locus. *pB42AD-DDI1-C276S-C300S *was constructed using the QuickChange site directed mutagenesis kit (Stratagene) using the following mutagenesis primers: DDIC300S-1 5'CTGAAAAGGCATTGGCTAGTGTGGACTTAAAGGAAAAC3', DDIC300S-2 5'GTTTTCCTTTAAGTCCACACTAGCCAAATGCCTTTTCAG3', DDIC276S-1 5'AAAATAGAAACACAATATATTCCAAGCAGTTTTACCGTCTTAGATACTG3' and DDIC276S-2 5'CAGTATCTAAGACGGTAAAACTGCTTGGAATATATTGTGTTTCTATTTT3'.

DSK2 was disrupted by homologous recombination using a fragment obtained by PCR from the FA6 cassette using the primers: dsk2kan-5' (5'-ATAAGACGGATCAAAGACACCGAATCATTCTAGCACGATACAGCTGAAGCTTCGTACGCT-3') and dsk2kan-3' (5'-TAGGGTAAAAGTATATAGGTTGCGGCATCTAGACGTTTATGCATAGGCCACTAGTGGATC-3')

### Time courses

Yeast strains were grown in rich YEPD medium containing extra adenine overnight and diluted 1:20 before the experiment. For G1-release, diluted cultures were incubated for ~2 hours in the presence of 0.2 μg/mL alpha-factor (Sigma), then washed twice and released in fresh medium at 37°C. For nocodazole release, an alpha-factor arrest was performed first and cells were released into fresh medium at 37°C containing 15 μg/mL Nocodazole for 11/2 hours. Cells were washed twice with pre-warmed sterile water and released into pre-warmed rich medium containing 0.4 μg/mL alpha-factor to stop the cells from entering the next cell cycle. Alpha-factor was also added to the nocodazole-arrest medium 1/2 hour before releasing. At least 100 cells were counted per timepoint.

### Western blots

Whole cell lysates were separated by SDS-PAGE (10% acrylamide w/v), transferred to an Immobilon PVDF membrane and probed using rabbit polyclonal anti-Ddi1 1:5000, or rabbit polyclonal anti-Rad23 1:5000 [22].

## List of Abbreviations

GFP – Green Fluorescent Protein

SPB – Spindle Pole Body

UBA – Ubiquitin Associated

UBL – Ubiquitin Like

YEPD – Yeast Peptone Dextrose

## Competing interests

The author(s) declare that they have no competing interests.

## Authors' contributions

Experiments performed by LADM, YK. Experiment design by KJW and DJC.

## References

[B1] Rubin DM, Finley D (1995). Proteolysis. The proteasome: a protein-degrading organelle?. Curr Biol.

[B2] Reed SI (2003). Ratchets and clocks: the cell cycle, ubiquitylation and protein turnover. Nat Rev Mol Cell Biol.

[B3] Aguilar RC, Wendland B (2003). Ubiquitin: not just for proteasomes anymore. Curr Opin Cell Biol.

[B4] Sweder K, Madura K (2002). Regulation of repair by the 26S proteasome. J Biomed Biotechnol.

[B5] Schmidt M, Hanna J, Elsasser S, Finley D (2005). Proteasome-associated proteins: regulation of a proteolytic machine. Biol Chem.

[B6] Chen L, Madura K (2002). Rad23 promotes the targeting of proteolytic substrates to the proteasome. Mol Cell Biol.

[B7] Rao H, Sastry A (2002). Recognition of specific ubiquitin conjugates is important for the proteolytic functions of the ubiquitin-associated domain proteins Dsk2 and Rad23. J Biol Chem.

[B8] Madura K (2002). The ubiquitin-associated (UBA) domain: on the path from prudence to prurience. Cell Cycle.

[B9] Hartmann-Petersen R, Seeger M, Gordon C (2003). Transferring substrates to the 26S proteasome. Trends Biochem Sci.

[B10] Madura K (2004). Rad23 and Rpn10: perennial wallflowers join the melee. Trends Biochem Sci.

[B11] Elsasser S, Finley D (2005). Delivery of ubiquitinated substrates to protein-unfolding machines. Nat Cell Biol.

[B12] Funakoshi M, Sasaki T, Nishimoto T, Kobayashi H (2002). Budding yeast Dsk2p is a polyubiquitin-binding protein that can interact with the proteasome. Proc Natl Acad Sci USA.

[B13] Lambertson D, Chen L, Madura K (2003). Investigating the importance of proteasome-interaction for Rad23 function. Curr Genet.

[B14] Elsasser S, Gali RR, Schwickart M, Larsen CN, Leggett DS, Muller B, Feng MT, Tubing F, Dittmar GA, Finley D (2002). Proteasome subunit Rpn1 binds ubiquitin-like protein domains. Nat Cell Biol.

[B15] Saeki Y, Sone T, Toh-e A, Yokosawa H (2002). Identification of ubiquitin-like protein-binding subunits of the 26S proteasome. Biochem Biophys Res Commun.

[B16] Chen L, Shinde U, Ortolan TG, Madura K (2001). Ubiquitin-associated (UBA) domains in Rad23 bind ubiquitin and promote inhibition of multi-ubiquitin chain assembly. EMBO Rep.

[B17] Bertolaet BL, Clarke DJ, Wolff M, Watson MH, Henze M, Divita G, Reed SI (2001). UBA domains of DNA damage-inducible proteins interact with ubiquitin. Nat Struct Biol.

[B18] Wilkinson CR, Seeger M, Hartmann-Petersen R, Stone M, Wallace M, Semple C, Gordon C (2001). Proteins containing the UBA domain are able to bind to multi-ubiquitin chains. Nat Cell Biol.

[B19] Glockzin S, Ogi FX, Hengstermann A, Scheffner M, Blattner C (2003). Involvement of the DNA repair protein hHR23 in p53 degradation. Mol Cell Biol.

[B20] Elsasser S, Chandler-Militello D, Mueller B, Hanna J, Finley D (2004). Rad23 and Rpn10 serve as alternative ubiquitin receptors for the proteasome. J Biol Chem.

[B21] Saeki Y, Saitoh A, Toh-e A, Yokosawa H (2002). Ubiquitin-like proteins and Rpn10 play cooperative roles in ubiquitin-dependent proteolysis. Biochem Biophys Res Commun.

[B22] Bertolaet BL, Clarke DJ, Wolff M, Watson MH, Henze M, Divita G, Reed SI (2001). UBA domains mediate protein-protein interactions between two DNA damage-inducible proteins. J Mol Biol.

[B23] Kang Y, Vossler RA, Diaz-Martinez LA, Winter NS, Clarke DJ, Walters KJ (2006). UBL/UBA Ubiquitin Receptor Proteins Bind a Common Tetraubiquitin Chain. J Mol Biol.

[B24] Sasaki T, Funakoshi M, Endicott JA, Kobayashi H (2005). Budding yeast Dsk2 protein forms a homodimer via its C-terminal UBA domain. Biochem Biophys Res Commun.

[B25] Lowe ED, Hasan N, Trempe JF, Fonso L, Noble ME, Endicott JA, Johnson LN, Brown NR (2006). Structures of the Dsk2 UBL and UBA domains and their complex. Acta Crystallogr D Biol Crystallogr.

[B26] Rasnick D, Duesberg PH (1999). How aneuploidy affects metabolic control and causes cancer. Biochem J.

[B27] Duesberg P, Li R, Fabarius A, Hehlmann R (2005). The chromosomal basis of cancer. Cell Oncol.

[B28] Biggins S, Ivanovska I, Rose MD (1996). Yeast ubiquitin-like genes are involved in duplication of the microtubule organizing center. J Cell Biol.

[B29] Ivantsiv Y, Kaplun L, Tzirkin-Goldin R, Shabek N, Raveh D (2006). Unique role for the UbL-UbA protein Ddi1 in turnover of SCFUfo1 complexes. Mol Cell Biol.

[B30] Kaplun L, Tzirkin R, Bakhrat A, Shabek N, Ivantsiv Y, Raveh D (2005). The DNA damage-inducible UbL-UbA protein Ddi1 participates in Mec1-mediated degradation of Ho endonuclease. Mol Cell Biol.

[B31] Clarke DJ, Mondesert G, Segal M, Bertolaet BL, Jensen S, Wolff M, Henze M, Reed SI (2001). Dosage suppressors of pds1 implicate ubiquitin-associated domains in checkpoint control. Mol Cell Biol.

[B32] Wang Q, Goh AM, Howley PM, Walters KJ (2003). Ubiquitin recognition by the DNA repair protein hHR23a. Biochemistry.

[B33] Withers WE, Jowett JB, Stewart SA, Xie YM, Garfinkel A, Shibagaki Y, Chow SA, Shah N, Hanaoka F, Sawitz DG (1997). Human immunodeficiency virus type 1 Vpr interacts with HHR23A, a cellular protein implicated in nucleotide excision DNA repair. J Virol.

[B34] Heessen S, Masucci MG, Dantuma NP (2005). The UBA2 domain functions as an intrinsic stabilization signal that protects Rad23 from proteasomal degradation. Mol Cell.

[B35] Wolfe KH, Shields DC (1997). Molecular evidence for an ancient duplication of the entire yeast genome. Nature.

[B36] Walters KJ, Lech PJ, Goh AM, Wang Q, Howley PM (2003). DNA-repair protein hHR23a alters its protein structure upon binding proteasomal subunit S5a. Proc Natl Acad Sci USA.

[B37] Krylov DM, Koonin EV (2001). A novel family of predicted retroviral-like aspartyl proteases with a possible key role in eukaryotic cell cycle control. Curr Biol.

[B38] Richardson HE, Wittenberg C, Cross FR, Reed SI (1989). An essential G1 function for cyclin-like proteins in yeast. Cell.

[B39] Burke D, Dawson D, Stearns T (2000). Methods in Yeast Genetics: a Cold Spring Harbor Laboratory Course Manual.

[B40] Straight AF, Marshall WF, Sedat JW, Murray AW (1997). Mitosis in living budding yeast: anaphase A but no metaphase plate. Science.

[B41] Segal M, Clarke DJ, Reed SI (1998). Clb5-associated kinase activity is required early in the spindle pathway for correct preanaphase nuclear positioning in Saccharomyces cerevisiae. J Cell Biol.

